# Factors Associated with Low Screening Participation and Late Presentation of Cancer amongst Women in the Pacific Island Countries and Territories: A Systematic Review

**DOI:** 10.31557/APJCP.2021.22.5.1451

**Published:** 2021-05

**Authors:** Carol Kartika Naidu, Nicola Wiseman, Neil Harris

**Affiliations:** *School of Medicine, Australia. *

**Keywords:** Pacific Island, neoplasms, mass screening, participation, women, early detection of cancer

## Abstract

**Background and Objective:**

In most Pacific Island Countries and Territories (PICTs), cancer patients commonly present at very late stages and by the time the disease is diagnosed, it is often too late for treatment. This review examines the evidence on factors associated with low cancer screening participation and late presentation of cancer among women of the PICTs.

**Materials and Methods:**

Medline, PubMed, ProQuest and The Cumulative Index to Nursing and Allied Health Literature were searched to identify relevant studies for this review. Terms of medical subject headings was performed in combination with other key words such as “screening”, “delay”, “determinants”, “awareness”.

**Results:**

Eleven studies met the inclusion criteria of this review. Six factors were identified from these studies: resources and facilities, trust in the health care system, culture and tradition, modesty, awareness and socioeconomic status.

**Conclusion:**

Due to several barriers and factors, women in the PICTs are hindered from accessing cancer screening practices and often present late with cancer symptoms leading to advanced stage diagnosis. The findings of this study provide a foundation for future studies that could focus more in-depth to explain how these factors contribute to the presentation of cancer in late stages.

## Introduction

Worldwide, breast cancer and cervical cancer contribute substantially to the burden of disease experienced by women, both being significantly more prevalent and deadly compared to other types of cancer (Boyle and Levin, 2008; Akram et al., 2017). These cancers remain the leading cause of mortality amongst the female population despite astonishing advancements that have been made in treating these diseases over the last two decades (Akram et al., 2017). 

The Pacific Island Countries and Territories (PICTs) is a region that consists of 22 countries and territories and is highly diversified in terms of its geography, language, culture and socioeconomic status (Sarfati et al., 2019). Despite its uniqueness, PICTs still have common features such as fragile economies, poorly developed health systems and the high burden of cancer incidences (Sarfati et al., 2019). Over the last three decades, a good deal of research on non-communicable diseases has been conducted throughout the South Pacific, however, research on cancer is low (Foliaki et al., 2011). Awareness and surveillance of cancer in the PICTs is poor compared to neighbouring countries such as Australia (Olver et al., 2011) and USA (Siegel et al., 2016).

In most PICTs, cancer patients commonly present at very late stages and by the time the disease is diagnosed, it is often too late for treatment (Foliaki et al., 2017). This situation is of particular note given breast and cervical cancer in particular have a better prognosis if detected and treated at an early stage (Ajekigbe, 1991). Non-participation in screening services is often cited as the reason late presentation is so high (Lyimo and Beran, 2012). Projections into the future suggest that if no change in cancer incidence occurs, then the Western Pacific Region mortality linked with cancers will increase by an astounding 73.1% in the next 20 years (Cancer tomorrow, 2018). 

There remains limited understanding as to why cancer screening rates remain so low in the PICTs (McMullin et al., 2008). Often, a hindrance to early detection is a lack of awareness of available resources. This is evident in countries such as American Samoa, in which methods of detection are available or free, yet, detection and screening rates remain low (Mishra et al., 2001). Mishra and colleagues suggest that women often face embarrassment and modesty issues which serve as a major barrier to screening participation, especially in countries that are rich in culture, tradition and religion (Mishra et al., 2001). Barriers related to low screening participation and late diagnosis reflect the iceberg theory (Jeyarajasekar and Sivakumar, 2020), which suggests that the factors have a deeper meaning which may not be immediately evident and there is still a gap in understanding how these factors interplay and contribute to the current situation.

Cancer screening participation is commonly perceived as being dependent on good health-seeking behaviours and when this is poor, for example not knowing the facilities or overlooking symptoms, then a blame game is played where the fault rests with the patients (Awofeso et al., 2018). However, it is essential that other factors that could contribute to successful early diagnosis rates are understood and addressed (Awofeso et al., 2018). Barriers and factors that contribute to low screening participation and late presentation of cancer can vary from country to country and no single factor can fit all. This review examines the evidence on factors associated with low cancer screening participation and late presentation of cancer among women of the PICTs.

## Materials and Methods

*Search Strategy*

Medline, PubMed, ProQuest and The Cumulative Index to Nursing and Allied Health Literature (CINAHL) were searched to identify relevant studies for this review. There was no date restriction, however, to be included in the review a paper needed to be English-language and published in a peer-reviewed journal. Table 1 presents an example of a detailed search strategy that was used to identify papers that were relevant on Medline. Searches for Medical Subject Headings (MeSH) were also conducted on Medline. Similar search strings were used on PubMed, ProQuest and CINAHL. 

*Selection Criteria*

The review included studies if they were published in English-language peer-reviewed journals, reported factors associated with low participation in cancer screening amongst women, reported factors associated with late presentation of cancer among women, presented data separately on women and men, presented data on any types of cancer, conducted the study in the three major groups of islands in the Pacific Ocean; Melanesia, Micronesia and Polynesia and conducted the research in Fiji or Papua New Guinea or Solomon Islands or Vanuatu or New Caledonia or Caroline Islands or Gilbert Islands or Mariana Islands or Marshall Islands or Easter Island or Samoa or Tonga or Cook Islands or Tuvalu or Tokelau or Niue or Wallis and Futuna or French Polynesia or Kiribati or Palau or Guam. The review excluded studies if they were not studying that were original; conference abstracts, opinions, editorials, focused on general screening and not specifically screening for women, were studies conducted in Hawaii and/or New Zealand, only reported findings in developed countries and conducted studies on pacific islanders who reside in America, New Zealand, Australia or any country apart from those included in the study. The inclusion and exclusion criteria were used to initially screen the studies using their titles and abstracts. This process was then followed by selecting relevant articles by full-text screening.

*Quality Assessment*

The studies were assessed for quality using a scoring system adapted from Johannesen and LoGiu-dice (2013). The quality assessment scoring criteria enabled assessment as to whether the studies had clearly defined the inclusion and exclusion criteria, minimised selection bias through sampling (for instance, through the use of randomisation or similar technique), had a good response rate (i.e. ≥80%), defined the study outcome, used valid and reliable instruments to measure the study outcome, defined the risk factors, used valid and reliable instruments to measure any risk factors and adjusted for confounding risk factors. One point was assigned for each category and the scores were totaled for each study included in this review (see Table 2).

*Data Extraction*

The extraction of the data was independently performed by one author (CKN) using a data extraction template and subsequently checked by a second and third author (NH, NW). The extraction template was inclusive of the first author, date of publication, journal in which it was published, response rate, type of screening, sample size, geographical location, conceptual framework, factors, sampling method, study setting, age of participants and associated factors. Factors that were presented in each of the papers were analysed and grouped together according to its context and similarity. All the factors were then collated into six overall factors. Factors were extracted based on its method of information collection and whether it was information obtained from the research in the paper. (Table 2).

## Results

**Figure 1:**
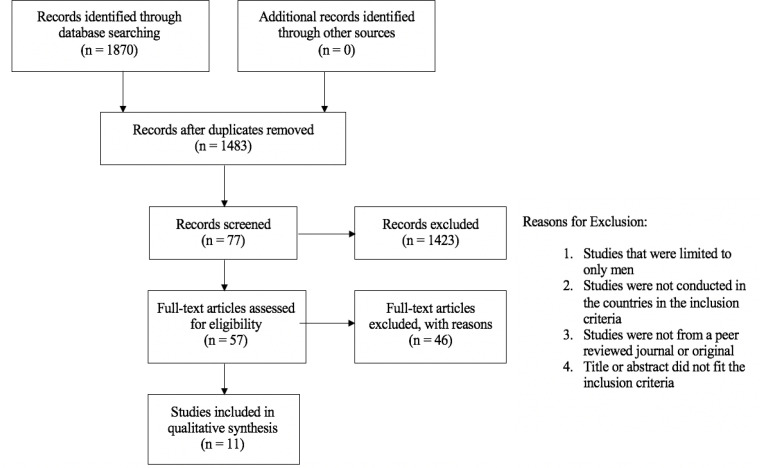
PRISMA Flow Diagram Showing the Process of Article Filtration (PRISMA, 2009)

Initially, a total of 1870 papers were identified for this review and were retrieved from four databases. The papers were screened and included based on titles and abstracts followed by the removal of duplicate papers and then a more detailed review of papers (see Figure 1). In total, 11 papers were included in this review (see Table 2). 

*Study Characteristics*

Table 2 overviews the 11 studies that were included in this review. The reviewed studies presented evidence of factors associated with low participation in cancer screening services and late presentation of cancer among women of nine countries (see table 2). Two studies focus on breast cancer (SenGupta et al., 1990; Pape et al., 2016), five studies focussed on cervical cancer (Fotinatos et al., 2010; Rius et al., 2013; Fong et al., 2014; Kelly-Hanku et al., 2018; Van Dyne et al., 2010) and four studies focused on both (Ou et al., 2004; Tseng et al., 2004; Tsark et al., 2007; Tutti et al., 2017). Most of the papers focused specifically on one geographical location, however, one study explored six US associated regions, three of which were of interest to this study; Republic of the Marshall Islands, Commonwealth of the Northern Mariana Islands, Federated States of Micronesia.

The sample size of the included studies ranged from 45 to 3500 participants, with the age of participants ranging from 10. One study used the framework of local biology “to explore the production of unstable biomedical knowledge of cervical cancer in PNG” (Tsark et al., 2007), while 10 of the remaining studies had no specific theoretical framework mentioned. Regarding the quality of the included studies, five studies achieved a score of three or more while six studies achieved a score of two or less out of a possible eight. Most studies did not use reliable and measurable instruments to define factors and barriers. Five studies were not cross-sectional studies and were epidemiological studies that used secondary data from hospital records instead (SenGupta et al., 1990; Rius et al., 2013; Ou et al., 2004; Tseng et al., 2004; Van Dyne et al., 2010; Tutti et al., 2017). 

The included studies focussed on screening participation related to several screening methods. Three studies used a mammogram for breast cancer screening (Tseng et al., 2004; Pape et al., 2016; Tutti et al., 2017), five studies used pap smear (Fotinatos et al., 2010; Rius et al., 2013; Tseng et al., 2004; Tsark et al., 2007; Tutti et al., 2017) and two studies used visual inspection of the cervix with acetic acid for cervical cancer screening (Fong et al., 2014; Tsark et al., 2007), one study used physical breast examination for breast cancer screening (Tsark et al., 2007). Three studies did not mention the type of screening used (SenGupta et al., 1990; Ou et al., 2004; Van Dyne et al., 2010). A range of assessment techniques was used to examine factors related to screening participation and/or late presentation. Techniques included looking at whether the factors were derived during the course of the research through surveys, questionnaires, cancer registries and patient records.

*Factors Associated with low screening participation and late presentation of cancer in women*

Factors reported by the reviewed studies as being associated with low screening participation and/or late presentation of cancer in women are presented in Table 2. Six factors were identified and include; resources and facilities (SenGupta et al., 1990; Ou et al., 2004; Tseng et al., 2004; Fong et al., 2014; Pape et al., 2016; Tsark et al., 2007; Van Dyne et al., 2010; Tutti et al., 2017), trust in the health care system (Tseng et al., 2004), culture and tradition (SenGupta et al., 1990; Fong et al., 2014; Pape et al., 2016; Tsark et al., 2007), modesty (Fotinatos et al., 2010; Tseng et al., 2004; Tutti et al., 2017), awareness (SenGupta et al., 1990; Fotinatos et al., 2010; Rius et al., 2013; Tseng et al., 2004; Pape et al., 2016; Tsark et al., 2007; Tutti et al., 2017), and socioeconomic status (Fong et al., 2014; Pape et al., 2016). A description of each of these factors is presented in the following sections. 

*Resources and Facilities*

Eight of the included studies identified resources and facilities as a barrier to low screening participation (Ou et al., 2004; Tseng et al., 2004; Fong et al., 2014; Pape et al., 2016; Tsark et al., 2007; Van Dyne et al., 2010; Tutti et al., 2017), one of these studies identified the same barrier for late presentation (SenGupta et al., 1990). A study in Papua New Guinea (PNG), associated a lack of resources and facilities to late presentation of breast cancer, where most patients presented at late stages with fungating masses and ulceration (SenGupta et al., 1990). Three studies identified that limited patient access to different levels of health care leads to delays in screening, diagnosis and treatment (Ou et al., 2004; Tsark et al., 2007; Van Dyne et al., 2010). For example, three Federated States of Micronesia states do not have mammography units and those that do limit usage for diagnostic purposes rather than for breast cancer screening (Tsark et al., 2007). It was noted some countries did not have the equipment to carry out tests and screenings with some procedures needing to be referred or diagnosed overseas. A study that included Marshall Islands, stated remoteness and limited on island treatments contribute towards the delayed presentation of cancer as many women have to travel to main islands for health care and screening services (Tsark et al., 2007). Four studies agreed that geography contributes to a lack of patient access to screening services, which is then attributed to transportation barriers (Ou et al., 2004; Tseng et al., 2004; Fong et al., 2014; Pape et al., 2016). A study in Palau noted that low screening rates may be attributed to maintenance issues of screening equipment which may leave a screening device unusable for extended periods (Tutti et al., 2017). 

*Trust in The Health Care System*

One study conducted in the Commonwealth of The Northern Mariana Islands identified trust in the health care system as a barrier to screening participation (Tseng et al., 2004). The study reported that “distrust of the healthcare system” was one of the many reasons for low screening participation rates amongst women. The study did not explain of the factor. 

*Culture and Tradition*

Four of the included studies identified culture and tradition as a factor contributing to low screening participation (SenGupta et al., 1990; Rius et al., 2013; Fong et al., 2014; Pape et al., 2016). One study identified the same factor for late presentation (SenGupta et al., 1990). A study in PNG stated that many Melanesians have a reluctance to die outside tribal boundaries, however, this statement was not elaborated adequately as to why it is a direct factor relating to the late presentation and low screening participation of breast cancer (SenGupta et al., 1990). A study conducted in Easter Island noted that awareness is low in Aboriginal Polynesians, most likely due to cultural barriers when compared to the population of Chile, who showed comparatively higher screening participation rates perhaps due to higher exposure to education (Rius et al., 2013). Two studies reported that low screening participation was most likely attributed to culture, tradition and societal practices (Fong et al., 2014; Pape et al., 2016). For example, in PNG the nature of mammography services contributes towards the unwillingness of a patient to engage in the service, with a community member quote provided to support this assertion: “The culture in my area does not allow any males to touch any part of a female’s body” (Pape et al., 2016).

*Modesty*

Three of the included studies identified modesty as a barrier to screening participation (Fotinatos et al., 2010; Tseng et al., 2004; Tutti et al., 2017). The studies reported that “embarrassment” and “embarrassment of gynaecological exams” were some of the many reasons there are low screening participation rates amongst women (Fotinatos et al., 2010; Tseng et al., 2004). Two studies were conducted in Vanuatu and Commonwealth of The Northern Mariana Islands (Fotinatos et al., 2010; Tseng et al., 2004). The study conducted in the Commonwealth of The Northern Mariana Islands reported that women felt that in small communities there was a loss of privacy during pap smears and mammograms as most clinicians are not females (Tseng et al., 2004). In Palau, low screening rates were, at least in part, explained as being due to women feeling uncomfortable during breast and cervical cancer examinations, due to its personal nature (Tutti et al., 2017). 

*Awareness*

Awareness was identified as a barrier to cancer screening participation in seven studies (SenGupta et al., 1990; Fotinatos et al., 2010; Rius et al., 2013; Tseng et al., 2004; Pape et al., 2016; Van Dyne et al., 2010; Tutti et al., 2017). Three studies reported that low screening participation is often due to the lack of awareness about cancer symptoms and screening services (Fotinatos et al., 2010; Rius et al., 2013; Tseng et al., 2004). A study in the Marshall Islands reported that low awareness is often a reason which contributes towards low screening, thereby reducing the likelihood of early detection of cancer (Tsark et al., 2007). The paper authored by SenGupta identified not recognising the significance of cancer symptoms as a barrier to early presentation. The study was conducted in PNG and noted that “ignorance of patients” could be a reason why many patients present late with metastasized tumours (SenGupta et al., 1990). Three studies presented that a lack of education and knowledge about cancer and screening services are often associated with low participation in screening activities (Fotinatos et al., 2010; Rius et al., 2013; Pape et al., 2016). The study in Palau noted that women may be unaware of the benefits of early detection and hence not access the available screening services (Tutti et al., 2017). A study conducted in PNG highlighted that often lack of knowledge about a woman’s private parts and women-related issues are also reasons why women are late to attend health care and screening services (Tsark et al., 2007). 

*Socioeconomic Status*

Two of the included studies identified socioeconomic status as a factor or barrier to low screening participation (Fong et al., 2014; Pape et al., 2016). Both these studies noted that finance and employment played a crucial role in determining whether women would participate in screening services (Fong et al., 2014; Pape et al., 2016). A study conducted in Fiji argued that women living in rural areas of Fiji may not be as aware of the importance of cervical cancer screening due to not having paid employment as opposed to those that live in more urban locations and have jobs (Fong et al., 2014). 

**Table 1:** Search String (Medline)

1. Neoplasm* OR Cancer* OR Tumor* OR Tumour* OR Carcinoma Breast OR Mammary
2. (delay* OR late OR poor) AND (presentation OR attendance OR diagnosis OR stage OR detection OR prognosis) AND (Low Cancer Screening Participation) AND (Mammography OR “Pap Smear” OR “Visual inspection of the cervix with acetic acid” OR “Breast Examination” OR “Colonoscopy” OR “Sigmoidoscopy” OR “Stool Tests” OR “Low-dose helical computed tomography” OR “Alpha-fetoprotein blood Test” OR “Breast MRI” OR “CA-125 Test” OR “PSA Test” OR “Skin exams” OR “Transvaginal Ultrasound” OR “Virtual Colonoscopy”)
3. determinant* OR factor* OR reason* OR belie* OR awareness OR perception OR “Social Perception” OR opinion OR attitude OR “social value” OR “social norm” OR understand* OR language OR communicat* OR fear OR mistrust OR relig* OR knowledge OR barrier OR embarrass* OR income OR socioeconomic OR educat* OR poor OR poverty OR attitude
4. Fiji OR "Papua New Guinea" OR "Solomon Islands" OR Vanuatu OR "New Caledonia" OR “Samoa” OR “Tonga” OR “Cook Islands” OR “Tuvalu” OR “Tokelau” OR “Niue” OR “Wallis and Futuna” OR “French Polynesia” OR "Easter Island" OR “Caroline Islands” OR "Federated States of Micronesia" OR “Palau” OR “Gilbert Islands” OR “Kiribati” OR “Northern Mariana Islands” OR “Guam” OR “Marshall Island”
5. Inciden* OR Epidemiology OR Prevalence OR Occurrence OR "Mortality Rate" OR "Mortality" OR "Death Rate" OR "Death"

MeSH Terms: 1, (MH "Neoplasms+"); 2, (MH "Breast Neoplasms+"); 3, (MH "Delayed Diagnosis+")

**Table 2:** Summary of the 11 Included Studies about Screening Participation and Late Presentation of Cancer among Women in the PICTs

First Author/ Year	Geographical Location	Sampling Method	Sample Size	Age of Participants	Screening Methods	Type of Cancer	Factors	Quality Score
SenGupta, 1990	PNG	Reports from hospital	382	10 - > 60	Not Mentioned	Breast	Resources and facilities Culture and tradition Awareness	1
Fotinatos, 2010	Vanuatu	Home visits and health facility patients	422	18-65	Pap Smear	Cervical	Awareness Modesty	5
Rius, 2013	Easter Island	Patient Statistics	49	Not Mentioned	Pap Smear	Cervical	Awareness	1
Ou, 2004	Kiribati	Reports from Hospitals	142	Not mentioned	Not Mentioned	Breast and Cervical	Resources and Facilities	1
Tseng, 2004	Commonwealth of The Northern Mariana Islands	Reports from Hospitals	304	Not Mentioned	Pap Smear and Mammogram	Breast and Cervical	Awareness Resource and Facilities Modesty	1
Fong, 2014	Fiji	Recruitment of participants through awareness programs	1971	30-49	VIA	Cervical	Resource and Facilities Socioeconomic Status Culture and Tradition	4
Pape, 2016	PNG	Women with appointments at the hospital	3500	Not mentioned	Mammography	Breast	Resource and Facilities Awareness Socioeconomic Status Culture and Tradition	5
Tsark, 2007	Federated States of Micronesia	Assessment Tool	Not mentioned	Not Mentioned	Pap Smear and Breast Examination	Cervical and Breast	Resources and facilities Culture and Tradition	2
Kelly-Hanku, 2018	PNG	Semi-structured Interviews and Focus group discussion	57	15-59	VIA	Cervical	Awareness	4
Van Dyne, 2020	Republic of the Marshall Island	Reports from hospital	Not mentioned	Not mentioned	Not mentioned	Cervical	Resources and Facilities	1
Tutti, 2017	Palau	Reports from hospital	45	35-65	Pap Smear, Mammogram and physical breast examination	Breast and Cervical	Awareness, Resources and Facilities and Modesty	3

## Discussion

This review has gathered evidence of six factors associated with low participation in cancer screening services and late presentation of cancer among women of the PICTs. However, it must be kept in mind that there are differences across the 11 included studies in the type of measurements used in determining these factors and consequently, it is difficult to compare the influence of factors. 

Some studies included in the review noted that the participation rate and diagnosis of cancer remained low in the female population despite the establishment of in-country screening services (Tutti et al., 2017). This indicates that a substantial number of women are still not able to access and reach such screening and diagnostic health services. The study findings will inform the present gap in knowledge which could then prompt interventions which will be useful in promoting early detection of cancer. 

Lack of adequate healthcare resources and facilities was consistently reported to be a barrier to cancer screening participation in six studies and a factor for late diagnosis of cancer in one study. This is reflected with evidence that PICTs have inadequate screening, oncology and surgical services (Sarfati et al., 2019). A recent report published in The Asian-Pacific Resource and Research Centre for Women (ARROW), noted that in Fiji, there is a significant gap in the healthcare system and late presentation of cancer is highly prevalent. The report stated that accessibility to correct information and available treatment options are overly low (Fiji Women’s Rights movement, 2018). Countries such as PNG and the Federated States of Micronesia face difficulty in providing a range of screening services and in most cases screening tools are used for diagnostic purposes rather than being positioned within primary care to be used for screening purposes (SenGupta et al., 1990; Tsark et al., 2007). In Micronesian women, the cervical cancer incidence is about 8 times higher than the United States national average and is believed to be due to resource, educational and geographic barriers (Leon Guerrero et al., 2020). Due to the lack of health resources in the community, there are scenarios when women in the PICTs are unable to readily access screening services or get proper diagnosis unless they travel to the main islands (Tsark et al., 2007). This is consistent with previous studies where health-seeking behaviour and early detection of cancer was found to be dependent on the distance to the nearest hospital and the costs associated with the travel (Mwaka et al., 2015).

Association between culture and tradition and low participation in cancer screening services and late presentation of cancer were consistent throughout six studies and proved to be a strong finding in this review. Pape (2014) reported that women in PNG are reluctant to participate in mammography services due to the nature of this examination as it is forbidden for any other men, apart from the female’s husband, to touch a woman. Research suggests that it may be beneficial for health care systems to work alongside the religious ‘gatekeepers’ in the community (Bianco et al., 2017). Such collaboration could incite confidence in the traditional population and thereby improve participation in an environment that could prove to be comfortable and accepted by the wider community (Bianco et al., 2017). In doing so, the services and approach should reflect the values and beliefs of the population and cater to literacy levels, so that medical and health terminology is comprehended by the patients. Hence, any approach taken must be culturally tailored to the local community setting (Bianco et al., 2017). To explore the relative importance of culture it could be of interest to compare differences in health seeking behaviours of Pacific Islanders residing in more developed countries as opposed to those in less developed countries such as those included in this paper. 

Interestingly, Tseng et al., (2004), attributed lack of trust in the health care system as a reason for low screening participation. However, no other included studies reported this as a factor and there was insufficient detail provided to position this as a strong and compelling factor in this review. Nevertheless, it should be noted that being able to foster the patient-doctor relationship was key in establishing higher use of preventive services and reduced care disparity (Musa et al., 2009). 

Modesty was another theme that was recognized in this study. Specifically, embarrassment was identified as a factor under this theme and like other studies, women often associate screening activities with shame (Tseng et al., 2004; Fotinatos et al., 2010). For example, some women felt that a diagnosis of cervical cancer indicated sex outside of marriage and hence were ashamed of participating in screening services (Marlow et al., 2015). Cancer reflects “continual stigmatization” and women diagnosed with cancer often keep it a secret as many fear abandonments by family or their male partner (Bailey et al., 2000). Modesty has strong connections to cultural and traditional barriers where feelings of embarrassment are often incited by the belief system of a community. 

Overall, in many of the included studies, it was noted that having a lack of awareness about cancer screening practices and symptoms are often reasons why women do not seek health care services. In this review, awareness is presented as knowledge of cancer services, symptoms and education about cancer in general. Often due to lack of knowledge, an individual may fail to recognise symptoms which are significant to cancer, which in this review, was highlighted among women from PNG (SenGupta et al., 1990). To some extent, awareness is determined by a person’s level of education, and this review also found that a person’s lack of education meant lower awareness (Fotinatos et al., 2010). A study notably mentioned “information gap leading to the fear of unknown”, which manifests as fear of cancer in general, hence, creating fear of cancer diagnosis, procedures and surgeries (Jain et al., 2016). The findings of this review translate to the need of increasing awareness and targeted education to ensure promptness of early detection through high screening participation. To increase cancer awareness in women, awareness campaigns can be established with the emphasis on multimedia and encouragement by health professionals at all community levels within a hospital or any clinical setting. These campaigns could also incorporate messages which would aim to counter cultural and modesty barriers. To further reduce the cancer burden, efforts to improve population awareness should be coupled with investment in screening and diagnostic equipment and overall improvement of health care delivery (Awofeso et al., 2018).

Another factor seen as a barrier was the socioeconomic status of an individual. Several included studies reported that the financial, political and employment aspects of a female determined whether a woman was likely to attend a screening service. In line with what other studies have found, affordability is a barrier and screening services are usually not accessible by those individuals that are socially disadvantaged (Phaswana-Mafuya and Peltzer, 2018). As a result, a subset is singled out to be underprivileged and hence largely excluded from proper and adequate health care that could prevent late-stage diagnosis of cancer.

The findings of this study are subject to several limitations and the results should be interpreted with caution. First, the overall quality of the studies was assessed as low, with some studies being incidence and epidemiologically driven as opposed to cross-sectional studies which specifically report and collect data on the barriers. Second, the amount of research currently available on this topic is limited. Third, the inconsistent definition of barriers, late presentation and reporting of patient stage of diagnoses made it difficult to quantify and compare extent of delay or rate of screening. The stated limitations contribute towards difficulty in comparing the results and put this review in a difficult position to make recommendations for strategies that could lead to improved screening practices in the PICTs. 

In conclusion, the data presented in this study provides insight into the broad factors that influence participation in cancer screening services and late presentation among women in the PICTs. The findings of this study provide a foundation for future research that could focus on building more in-depth explanations of the factors that contribute to the low participation in cancer screening services and presentation of cancer in late stages for women living in the PICTs. 

## Author Contribution Statement

Conceptualization: CKN, NW, NH; Writing original draft manuscript: CKN; Data/reference curation: CKN; Preparation of revised manuscript: CKN, NW, NH; Review and submission of final manuscript: CKN, NW, NH.
